# Short-chain fatty acid level and field cancerization show opposing associations with enteroendocrine cell number and neuropilin expression in patients with colorectal adenoma

**DOI:** 10.1186/1476-4598-10-27

**Published:** 2011-03-14

**Authors:** Danny CW Yu, Jonathan P Bury, James Tiernan, Jennifer S Waby, Carolyn A Staton, Bernard M Corfe

**Affiliations:** 1 Department of Oncology, University of Sheffield, The Medical School, Beech Hill Road, Sheffield, S10 2JF, UK

## Abstract

**Background:**

Previous reports have suggested that the VEGF receptor neuropilin-1 (NRP-1) is expressed in a singly dispersed subpopulation of cells in the normal colonic epithelium, but that expression becomes dysregulated during colorectal carcinogenesis, with higher levels in tumour suggestive of a poor prognosis. We noted that the spatial distribution and morphology if NRP-1 expressing cells resembles that of enteroendocrine cells (EEC) which are altered in response to disease state including cancer and irritable bowel syndrome (IBS). We have shown that NRP-1 is down-regulated by butyrate in colon cancer cell lines *in vitro *and we hypothesized that butyrate produced in the lumen would have an analogous effect on the colon mucosa *in vivo*. Therefore we sought to investigate whether NRP-1 is expressed in EEC and how NRP-1 and EEC respond to butyrate and other short-chain fatty acids (SCFA - principally acetate and propionate). Additionally we sought to assess whether there is a field effect around adenomas.

**Methodology:**

Biopsies were collected at the mid-sigmoid, at the adenoma and at the contralateral wall (field) of 28 subjects during endoscopy. Samples were fixed for IHC and stained for either NRP-1 or for chromogranin A (CgA), a marker of EEC. Stool sampling was undertaken to assess individuals' butyrate, acetate and propionate levels.

**Result:**

NRP-1 expression was inversely related to SCFA concentration at the colon landmark (mid-sigmoid), but expression was lower and not related to SCFA concentration at the field. Likewise CgA^+ ^cell number was also inversely related to SCFA at the landmark, but was lower and unresponsive at the field. Crypt cellularity was unaltered by field effect. A colocalisation analysis showed only a small subset of NRP-1 localised with CgA. Adenomas showed extensive, weaker staining for NRP-1 which contrastingly correlated positively with butyrate level. Field effects cause this relationship to be lost. Adenoma tissue shows dissociation of the co-regulation of NRP-1 and EEC.

**Conclusion:**

NRP-1 is inversely associated with levels of butyrate and other SCFA *in vivo *and is expressed in a subset of CgA expressing cells. EEC number is related to butyrate level in the same way.

## Background

The incidence of colorectal cancer has been shown to be decreased in populations with a high dietary fibre intake [1,2]. This effect is thought be attributable in part to the cellular actions of butyrate, a short-chain fatty acid (SCFA) produced by fermentation of fibre and resistant starch in the human colon lumen [3]. Butyrate is thought to be a chemoprotective effector, inhibiting colon carcinogenesis through regulation of cell cycle, apoptosis and angiogenic pathways [1,4-6].

Our recent data show that the transmembrane glycoprotein neuropilin-1 (NRP-1) is downregulated by butyrate in several colon cancer cell lines [7]. NRP-1 was originally characterised as a neuronal semaphorin receptor [8,9] and has since been identified as a non-tyrosine kinase co-receptor for some isoforms of the vascular endothelial growth factor (VEGF) family, the most potent pro-angiogenic family identified to date [10]. Angiogenesis is essential for tumour development and is stimulated at the earliest stages of the adenoma-carcinoma sequence in the colon and correlates with an increase in VEGF expression [11]. NRP-1 is up-regulated not only in vessels within adenomas and carcinomas, but also in hyperplastic adenoma cells and invasive colon cancer compared to normal mucosa. Overexpression of NRP-1 in these contexts is thought to enhance cancer cell survival [12] leading to cancer progression, metastatic potential and potential chemoresistance [13]. Immunohistochemical analysis has also identified NRP-1 expression in a subset of singly dispersed colonic epithelial cells [13,14] interpreted as enteroendocrine cells (EEC). However, regulation of this expression in normal mucosa remains uncharacterised.

Enteroendocrine cells (EEC) are hormone-producing intestinal epithelial cells that are individually dispersed throughout the epithelium where they have a critical role in regulating gastrointestinal physiology [15]. The numbers of colonic EEC have been shown to alter in conditions including irritable bowel syndrome [16] and cancer [17,18]. Indeed, the numbers of chromagranin A (CgA)-expressing EEC was shown to be decreased in mucosa adjacent to colon tumours compared to normal mucosa [18], although the mechanisms regulating this change are currently unknown. EEC have been shown to express the G-protein coupled receptors, GPR41 and GPR43, for SCFA including butyrate, acetate and propionate [19], suggesting that these cells may mediate, at least in part, the colon epithelial response to SCFA.

Our recent data show an inverse causal relationship between butyrate concentration and NRP-1 expression at both the mRNA and protein level *in vitro *[7]. We hypothesize that this is a representative model of *in vivo *systems and the same relationship will occur in vivo. In the present study we have investigated the relationship between faecal butyrate, acetate and propionate concentration and NRP-1 expression in human colonic mucosa. Furthermore, as NRP-1 expression is limited in the normal mucosa and is widespread in cancer tissue, we sought to investigate the expression profile of NRP-1 in adenoma and in fields around adenoma to map the onset of NRP-1 dysregulation. We have undertaken the same analyses on EEC and sought to establish whether EEC are the NRP-1 expressing compartment of the colon mucosa.

## Results

### Subject demographics

A total of 28 subjects with adenoma were recruited for whom biopsies and faecal butyrate data were available. All subjects were included. Subjects had a mean age of 68.1 ± 10.1 yr and a mean BMI of 25.5 ± 3.4 kg/m^2^. The concentration range of faecal butyrate was 0.64-16.4 mM.

### Butyrate level does not correlate with human colon crypt cellularity

**Figure 1:**
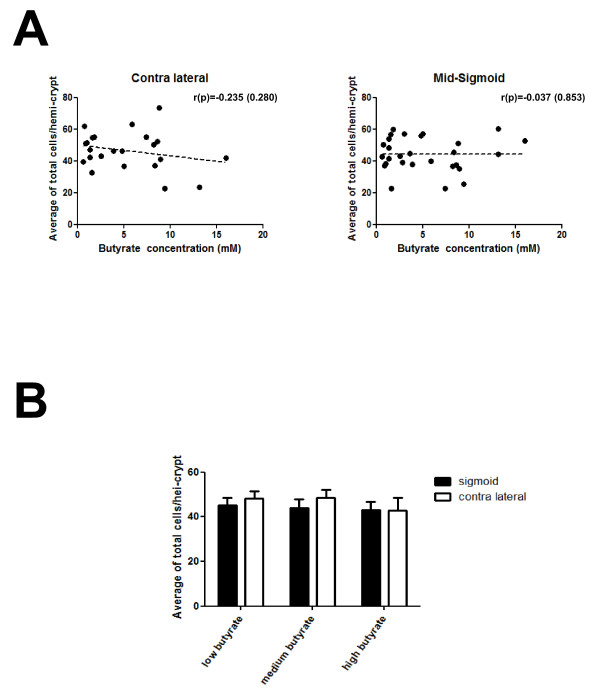
**Relationship between butyrate concentration and crypt cellularity**. The total number of cells per hemi-crypt was counted on 23 samples in contra lateral (field) and 28 samples in mid-sigmoid. (A) Graphs demonstrating that there was no relationship between cell number and butyrate at the field and mid-sigmoid. (B) The data were grouped into tertiles by butyrate concentration at high (> 8 mM), medium (2-8 mM) and low (< 2 mM) and presented as the mean ± SEM. The mean total cell number was 46.40/hemi-crypt in field tissue and 44.17/hemi-crypt in mid-sigmoid respectively showing no difference in crypt cellularity between field and mid-sigmoid samples.

In order to investigate any baseline associations between butyrate concentration and field effect on crypt cell number and to contextualize the main assessment of the impacts of these two factors on the proportion of NRP-1 and CgA positive cells, the total number of cells per hemi-crypt across 10 hemi-crypts per sample was determined on samples from 23 field and 28 mid-sigmoid specimens. The correlation between crypt cellularity and butyrate level was assessed by Spearman's rho (Figure 1A) and using Jonkheere-Terpstra (Figure 1B). These analyses show that there were no significant differences in crypt cellularity associated with butyrate level or associated with the adenoma field (Figure 1).

### Butyrate is associated with reduced NRP-1 protein expression in normal colon epithelial cells

**Figure 2:**
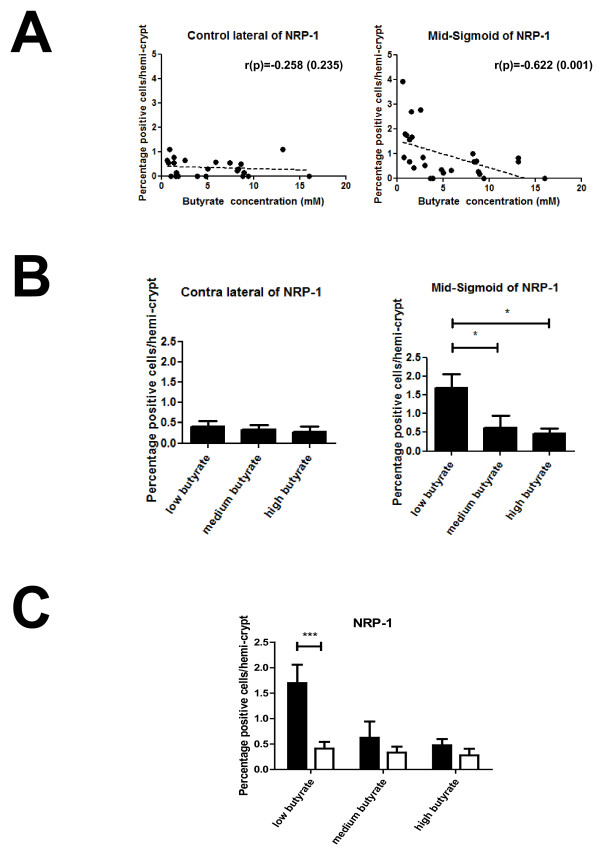
**NRP-1 protein expression in human colon epithelial cells**. The percentage of NRP-1 positive staining cells was calculated in crypts in the field and mid-sigmoid samples. (A) Graphs showing no relationship between butyrate concentration and NRP-1 expression in the field (contra-lateral) but a strong inverse correlation at the mid-sigmoid (r = -0.622; *p *= 0.001) (B) Graph showing the data as mean ± SEM when grouped into tertiles according to butyrate concentration. In the contra-lateral samples there were no differences seen, but in the mid-sigmoid a significant difference was seen between the low butyrate and mid/high butyrate groups (*p* < 0.015). (C) Graph comparing butyrate concentration and the percentage of NRP-1 expression in the contralateral (white bars) and mid-sigmoid (filled bars) samples. NRP-1 expression was significantly higher in the low butyrate group in the mid-sigmoid compared to the field (*p *= 0.003), but no other differences between sample sites were seen.

It has previously been reported that NRP-1 is expressed in singly dispersed cells within the colorectal epithelium [13,14] and is down-regulated by butyrate *in vitro *[7]. To investigate whether NRP-1 expression is associated with butyrate or other SCFA concentration in human non-malignant colonic epithelium, IHC staining was performed on 23 samples from the contralateral walls to the adenoma (fields) and 26 samples from mid-sigmoid of the same subjects (a constant landmark sampling point in all subjects). Only a small number of cells expressed NRP-1 in either the field (0.34% of crypt cells) or the mid-sigmoid (0.94% of crypt cells) specimens. There is a strongly negative correlation between butyrate concentration and NRP-1 positive cell count in mid-sigmoid (r = -0.622, *p* < 0.001; Figure 2 Table 1), however, this relationship was lost in the field (r = -0.258, *p *= 0.235; Figure 2 Table 1), where all crypts exhibit low numbers of NRP-1 positive cells (Figure 2A). Similarly when the data were grouped into tertiles by butyrate concentration: high (> 8 mM), medium (2-8 mM) and low (< 2 mM), there was a significant difference between groups at the mid-sigmoid landmark site (Jonkheere-Terpstra, *p *= 0.013). A *post hoc *analysis revealed that the NRP-1 positive cell count in the low butyrate group (1.69%) was significantly higher than in medium (0.62%, *p *= 0.016) and high (0.47%, *p *= 0.009) butyrate groups (Figure 2B). Interestingly, under conditions of low butyrate the percentage of NRP-1 expressing cells is significantly lower in the adenoma field compared to the landmark samples (*p *= 0.003, Figure 2C). Taken together, these data show that butyrate (> 8 mM) is associated with a reduction in the number of NRP-1 expressing cells in normal colorectal mucosa. Similar results were seen with acetate and propionate (see Table 1). This relationship is lost in the vicinity of adenoma, suggesting a field change in which normal regulatory mechanisms are suppressed.

**Table 1:** Correlations between SCFA and Np1 or CgA on adenoma specimens from Mid-sigmoid (MS) or contra-lateral wall (CL)

SCFA	Site	marker	Spearman's rho	p-value
**Butyrate**	MS	Np1 %	-0.622	0.001**
**Butyrate**	CL	Np1 %	-0.258	0.235
**Butyrate**	MS	CgA %	-0.370	0.053*
**Butyrate**	CL	CgA %	0	1.000
**Acetate**	MS	Np1 %	-0.653	0.001**
**Acetate**	CL	Np1 %	-0.214	0.328
**Acetate**	MS	CgA %	-0.224	0.251
**Acetate**	CL	CgA %	0.033	0.883
**Propionate**	MS	Np1 %	-0.555	0.003**
**Propionate**	CL	Np1 %	-0.144	0.511
**Propionate**	MS	CgA %	-0.130	0.511
**Propionate**	CL	CgA %	0.032	0.886
**Isobutyrate**	MS	Np1 %	-0.309	0.125
**Isobutyrate**	CL	Np1 %	-0.232	0.287
**Isobutyrate**	MS	CgA %	-0.224	0.251
**Isobutyrate**	CL	CgA %	-0.213	0.328

** highly significant; * approaching significance

### Butyrate is associated with reduced CgA expressing cell number in the colon epithelium

**Figure 3:**
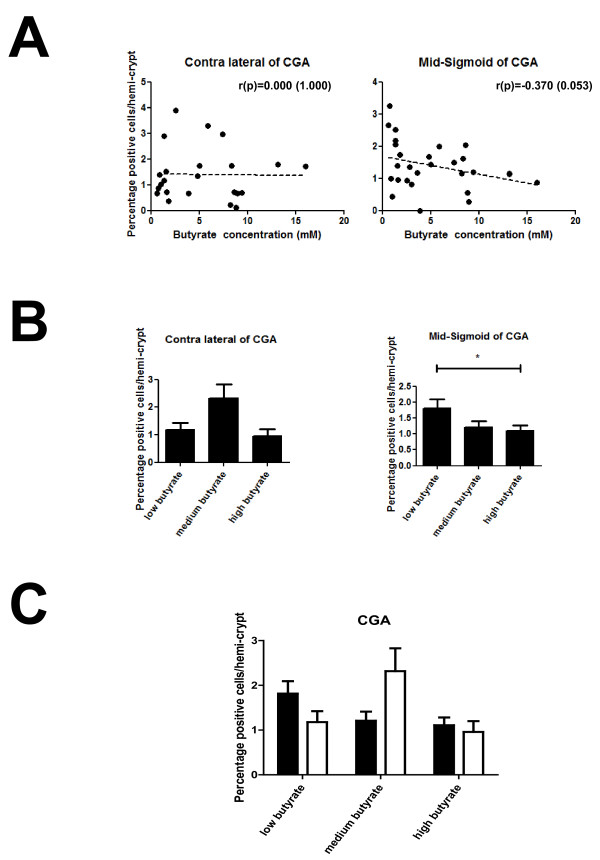
**CgA protein expression in human colon epithelial cells**. The percentage of CgA positive staining cells was calculated in crypts in the field and mid-sigmoid samples. (A) Graphs showing no relationship between butyrate concentration and CgA expression in the field (contra-lateral) but a moderate, almost significant inverse correlation at the mid-sigmoid (r = -0.370; *p *= 0.053) (B) Graph showing the data as mean ± SEM when grouped into tertiles according to butyrate concentration. In the contra-lateral samples there were no differences seen, but in the mid-sigmoid a significant difference was seen between the low butyrate and high butyrate groups (*p *= 0.037). (C) Graph comparing butyrate concentration and the percentage of CgA expression in the contralateral (white bars) and mid-sigmoid (filled bars) samples. No significant differences were seen between the field and mid-sigmoid samples in any groups.

The distribution of NRP-1 positive cells in normal colon epithelium mirrors that of enteroendocrine cells (EEC) [15]. The cell morphology of NRP-1 staining cells was also similar to that of EECs: relatively small nuclei and basally oriented cytoplasm, often without obvious continuity with the lumen. Moreover, EEC are known to express SCFA receptors [19]. Therefore in order to establish whether EEC number itself is associated with butyrate, acetate or propionate concentration in human normal colon tissue, IHC staining for CgA, a tissue marker for the majority of EEC subtypes [20], was undertaken on 23 samples in field and 28 samples in mid-sigmoid. CgA expression was observed in a small number of singly dispersed epithelial cells within the normal colon, up to 1.4% cells within a crypt (See Figure 6A). Spearman's rho analysis revealed a near-significant inverse correlation between the percentage of CgA expressing cells/crypt and butyrate concentration in the mid-sigmoid (landmark) samples (r = -0.370, *p *= 0.053; Figure 3 Table 1), but not in field samples (r = 0; *p *= 1.000; Figure 3 Table 1). When data were split into tertiles by butyrate level, the CgA positive cell fraction at low butyrate (1.82%) was higher than the medium (1.21%) and significantly higher than the high butyrate (1.11%) groups (*p *= 0.037) in mid-sigmoid sections (Figure 3B). There were no significant differences in the number of cells expressing CgA between field and mid-sigmoid samples or within fields, when grouped by butyrate level (Figure 3C). These data show that, as with NRP-1 expression, faecal butyrate concentration is associated with changes in endocrine cell numbers in normal human colon tissue, but that this relationship is flattened by field effects around adenoma. Relationships between EEC and acetate and propionate did not reach significance, although the direction of response to SCFA and field was as for butyrate (Table 1).

### NRP-1 expression only partly co-localizes with chromagranin A

**Figure 4:**
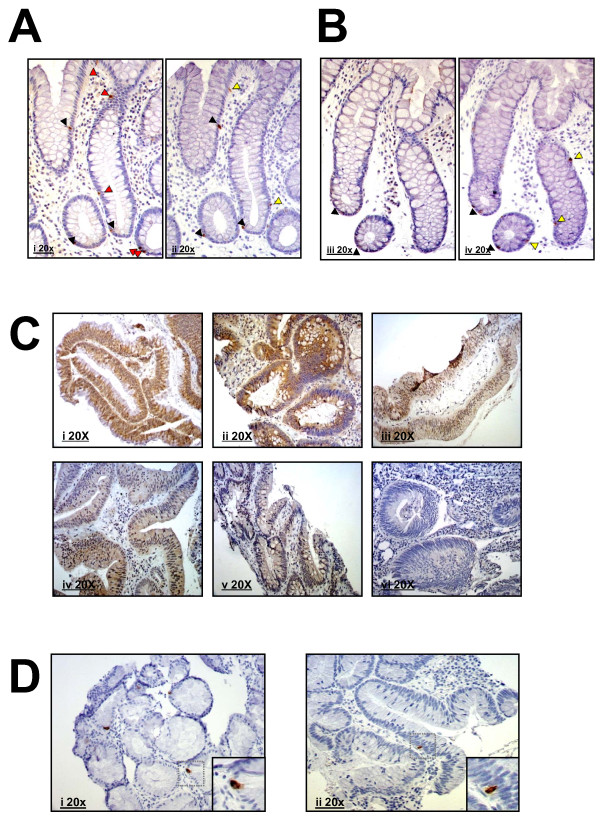
**Representative images of NRP-1 and CgA staining**. (A) Serial sections of mid-sigmoid samples and (B) contra-lateral (field) samples stained for NRP-1 (left) and CgA (right). Red arrow heads identify cells staining for NRP-1 only, black arrow heads identify cells staining for both NRP-1 and CgA and yellow arrow heads identify cells staining for CgA only. (C) Samples of adenoma demonstrating different intensities of staining and different percentage of cells staining for NRP-1 (D) Samples of adenoma showing only one or two cells staining for CgA.

**Figure 5:**
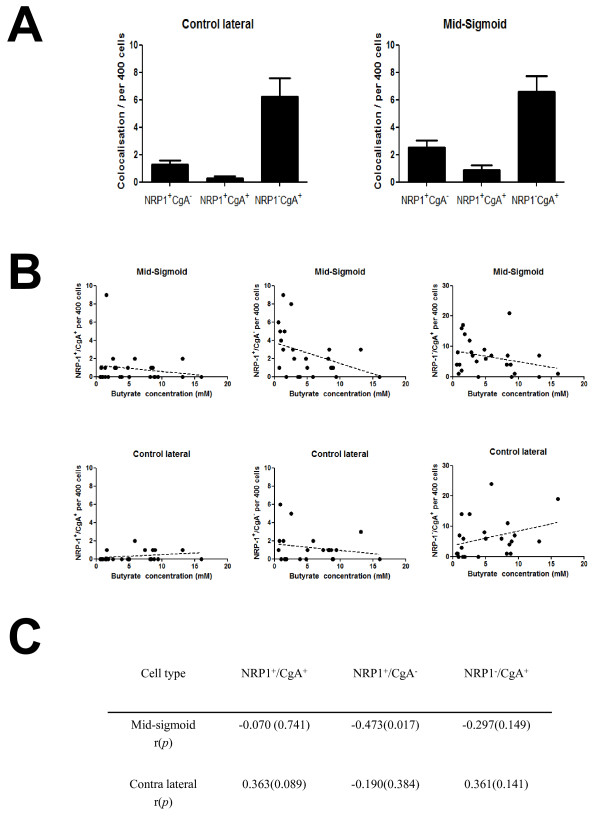
**Co-localisation of NRP-1 and CgA expression in human colon epithelial cells**. The co-localisation of NRP-1 and CgA expression was calculated in up to 400 cells in crypts in the field and mid-sigmoid samples. (A) Graphs showing of the relative abundance of NRP-1^+^/CgA^-^, NRP-1^+^/CgA^+ ^and NRP-1^-^/CgA^+ ^cells both in the field and mid-sigmoid and the data presented as the mean ± SEM (B) Graphs showing the relationships between NRP-1 and CgA colocalising and non-colocalising cells relative to butyrate level in the field and mid-sigmoid landmark. A significantly negative correlation was seen in the NRP1^+^/CgA^- ^cells in landmark (r = -0.473, p = 0.017). (C) The correlation was carried out by Spearman's statistics and the data were shown in r(p) value when grouped into NRP-1^+^/CgA^+^, NRP-1^+^/CgA^- ^and NRP-1^-^/CgA^+ ^in the field and landmark respectively.

In order to establish whether NRP-1 is expressed in the EEC compartment, adjacent sections stained for NRP-1 and CgA respectively were assessed for co-localisation (see Figure 4A). In both the field and landmark sites fewer than 10% of the CgA positive cells expressed NRP-1^+ ^and fewer than 20% of the NRP-1 positive cells expressed CgA^+ ^(Figure 5A). The levels of co-localisation did not alter between field and landmark sites (Figure 5A). Weak inverse correlations were seen between the number of NRP-1^+^/CgA^+ ^and NRP1^-^/CgA^+ ^cells at the mid-sigmoid site and butyrate levels and a significant inverse correlation was seen in the NRP-1^+^/CgA^- ^cells (r = -0.473; *p *= 0.017; Figure 4B). In contrast there were no significant correlations seen between butyrate and NRP-1^+^/CgA^+^, NRP-1^+^/CgA^- ^and NRP-1^-^/CgA^+ ^cells at the field site. Taken together these data show that NRP-1 expression does not predominantly co-localise with CgA expression.

### Butyrate is associated with increased NRP-1 protein expression in human polyp adenomas

**Figure 6:**
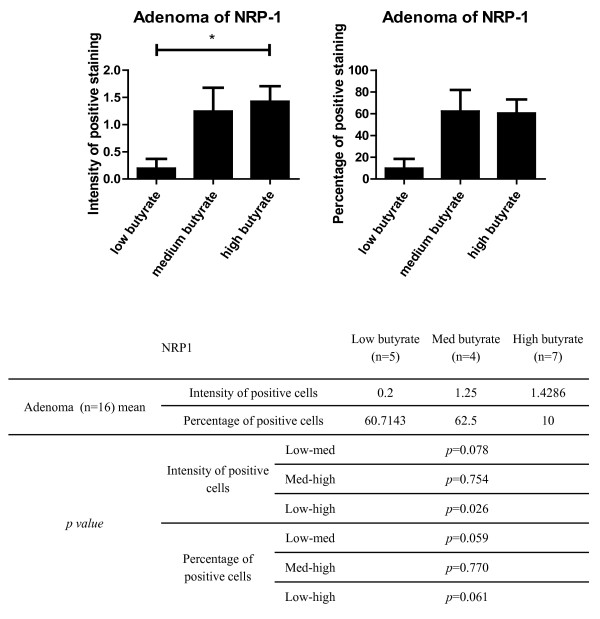
**NRP-1 protein expression in human polyps**. The expression of NRP-1 in human colorectal adenoma was characterised as intensity of stain (0-2; 0: no expression, 1: moderate expression and 2: strong expression) or the percentage of the adenoma cells expressing NRP-1. The data is presented as mean ± SEM and split into tertiles according to butyrate concentration. The graphs demonstrate lower NRP-1 expression correlates with lower butyrate level and a significant difference is seen between groups (Jonckeere-Terpstra; *p *= 0.026). *Post-hoc *analysis (Mann-Whitney U test) identified significance between the low and high butyrate groups with NRP-1 expression intensity, but not with the percentage of expressing NRP-1.

Previous studies have suggested that NRP-1 expression correlates with tumour growth and invasiveness in colorectal cancer [12] and that there is an increase in both intensity and area of expression from low-grade to high-grade dysplasia in colorectal adenomas [13]. Therefore in order to determine and confirm the expression pattern of NRP-1 in colon adenomas and to establish whether it is associated with butyrate concentration, IHC staining was performed on 16 human polyp samples from the same subjects. NRP-1 expression was generally expressed widely within the adenomas, albeit at a lower staining intensity than in cells in the normal mucosa (Compare Figure 4A and B with Figure 4C). NRP-1 staining was assessed, considering both staining intensity and the percentage of positively staining cells, using semi-quantitative scales. A strong positive correlation was seen between staining intensity and butyrate (r = 0.517; *p *= 0.040) and a near-significant correlation was seen between the percentage of positive cells and butyrate (r = 0.467; *p *= 0.053; Figure 6). When the data were grouped into tertiles by butyrate concentration there was a significant difference seen between the high and low butyrate groups (*p *= 0.026; Figure 6). Taken together these data suggest that NRP-1 expression is altered in adenomas and may be up-regulated by butyrate. Adjacent adenoma sections were concomitantly stained with CgA. In contrast with the NRP-1 staining pattern which altered markedly in adenoma by comparison with normal tissue, the CgA staining remained limited to singly dispersed cells (Figure 4D).

## Discussion

Previous studies have suggested that NRP-1 expression correlates with tumour growth and invasiveness in colorectal cancer [12] and that there is an increase in both intensity and area of expression from low-grade to high-grade dysplasia in colorectal adenomas [13]. The same studies have also reported that in the morphologically normal colon epithelium NRP-1 is expressed in a singly dispersed subpopulation of cells - with a distribution and frequency which we hypothesised might reflect localisation to EEC. Our recent data [7] show that butyrate, a product of fibre fermentation in the colon lumen, downregulates NRP-1 expression in colon cancer cell lines and we hypothesized that butyrate, and potentially other SCFA, produced in the lumen would have an analogous effect on the colon mucosa *in vivo*. We therefore sought to establish whether such a relationship exists *in vivo*.

Our studies confirm that NRP-1 is expressed in a subpopulation of individual dispersed cells within the colonic crypt epithelium, in agreement with previous data [13], and we observed that both the morphology and distribution of the NRP-1 cells resembled that of EECs. The expression of NRP-1 was inversely associated with faecal butyrate concentrations in agreement with our *in vitro *findings. Similar results were seen with acetate and propionate. These data suggest that the NRP-1 expressing cells are SCFA-responsive. Previous studies have identified SCFA receptors GPR41 and GPR43 on singly dispersed cells within the colonic mucosa [19] thought to be EEC. GPR41 was only expressed on 0.01 ± 0.01 cells/crypt and the staining was more frequent at the surface epithelium than the bottom of the crypt, unlike the staining seen for NRP-1. In contrast GPR43 was expressed in 0.33 ± 0.01 cells/crypt and was more evenly dispersed throughout the crypt i.e. similar to our staining for NRP-1 (an average of 0.37 ± 0.03 cells/crypt). GPR43 staining is specific to L-cells [21], which produce glucagon-like peptide-1 (GLP-1) and peptide YY (PYY) [22].

Although CgA is at present considered to be the broadest EEC marker, some EEC sub-populations are CgA negative [20]. The anti-CgA antibody used in this study has been reported as non-reactive in L-cells. As only a minority of CgA^+ ^EECs express NRP-1 and the majority of NRP-1 expression is out with the CgA^+ ^compartment, we hypothesize that NRP-1 is predominantly expressed in a different subset of EEC such as L-cells. It is notable that luminal non-digestible carbohydrates have been shown to modulate L-cell numbers in the rat [23]. As with the expression of NRP-1, there is an inverse relationship in the mid-sigmoid colon between CgA^+ ^cells and SCFA, albeit only significantly with butyrate. The relationship was lost in the field adjacent to adenoma in the same subjects. A previous study in xenograft mice has shown that the presence of a tumour (even distant to the intestine) depresses EEC cell number in the intestine [24], suggesting that a tumour expresses a diffusible factor which alters the normal regulatory mechanism for EEC number. Our data support this finding and show for the first time that EEC number is altered in the vicinity of a tumour in humans.

NRP-1 has been linked to cancer progression and aggressiveness in colon tumours [13]. Our data examining the expression pattern of NRP-1 and CgA in adenomas show that, whereas there are similarities and overlaps in morphologically normal tissue, the regulation of the two markers becomes profoundly unlinked in adenomas. The expression of CgA remains in singly dispersed cells (Figure 6D), as previously reported [18] and owing to the disorganised nature of the tissue and infrequent positive cells, scoring of the numbers of positives was not possible. In contrast NRP-1 expression was profoundly different to that seen in normal tissue. The staining in individual cells was generally lower in intensity, however it was no longer restricted to individual dispersed cells, but in many of the sections large areas stained positively, as seen in previous studies [13]. The pattern was scored for both intensity and proportion of positive staining and, in stark contrast to observations at the morphologically normal sites, showed a strong positive relationship with butyrate level. NRP-1 has been implicated as an anti-apoptotic protein in colon cancers [14] in addition to its role in angiogenesis [12], which is reinforced by its staining pattern not being limited to obvious microvessels. The staining pattern implicates NRP-1 as dysregulated early in adenomagenesis and it is likely that the role is, at least in part, anti-apoptotic in order to facilitate the growth and propagation of deranged tissue.

A plethora of *in vitro *studies using butyrate treatment of cell lines has shown that it induces apoptosis and cell cycle arrest at physiologically achievable concentrations (circa 1-10mM). Given that loss of regulation of both cell cycle and resistance to apoptosis are hallmarks of the cancer cell [25], these effects have been proposed as the key effectors of butyrate's hypothesized anti-neoplastic effect. However, these findings alone cannot explain the specificity of the effect and are offset by obverse findings regarding the cell cycle-promoting effects of butyrate on the normal colonocyte [26], leading to proposal of the "butyrate paradox" [27]. The implication of the butyrate paradox is that there may be a key change in early carcinogenesis which sensitizes the mutant colonocyte to normal levels of butyrate. However, Lupton [28,29] and our group [30] have asserted that some of the paradox can be explained by differences in experimental protocols and cellular and animal models used in different laboratories. The results herein suggest for the first time that opposing responses to butyrate can be seen in the normal and dysplastic colon. This implies a veracity in the paradox hypothesis in terms of altered response. However these data contradict the paradox hypothesis insofar as faecal butyrate levels associate with a factor, NRP-1, promoting poor prognosis rather than acting as a selective anti-neoplastic agent. As with folate, which protects against adenoma formation but which supports the growth of developed adenomas [31,32], butyrate (or more specifically the faecal stream) has been implicated in the continued growth of colorectal adenomas once formed [33]. These data support profound alterations in regulatory networks underpinning the earliest stages of adenoma formation and, as with folate, suggest caution is warranted in giving the same dietary advice for primary as for secondary chemoprevention.

One limitation of this study is the use of faecal SCFA as a proxy measure of luminal SCFA. There is a gradation of level of SCFA within the different regions of the colon lumen [34] but sampling luminal contents in humans remains a significant challenge to researchers in this field. Furthermore the SCFA level in itself may only represent a proxy measure of a further luminal metabolite with potentially stronger and causal effects on NRP-1 expression. As technologies for faecal metabonomics emerge, such possibilities may be explored in future.

Taken together our data provide evidence for progressive field effects in the vicinity of colon adenoma and in adenoma. Both NRP-1 and EEC number decreased in relation to increasing SCFA concentrations at sites distant to the adenoma. In the immediate field (our biopsy protocol sampled at the contra lateral wall) this apparent relationship to SCFA level is lost and in the neoplasia the effect is reversed with neuropilin showing positive association with butyrate and dissociated from EEC-like expression. Our data therefore suggest a progressive and complete reversal of the response to butyrate as a hallmark of field effects. Despite widespread acceptance of the field effect hypothesis [35-37] in colon carcinogenesis, we are aware of only one publication showing demonstrable molecular alterations at fields [38] highlighting the need to pursue this area of study more.

## Conclusion

In summary our data show that NRP-1 is expressed in the normal colon epithelium in a pattern redolent of EEC, and this expression appears related to butyrate levels, in agreement with the hypothesis raised from our *in vitro *data. NRP-1 expression is related to SCFA expression, but this association is lost in fields and expression becomes unlinked from EEC-like patterns in adenomatous tissue, implying an early and potential alternative role for NRP-1 in neoplasia. Our data showed for the first time that EEC number is also related to butyrate concentration. Future studies will now address whether there is a difference in EEC number in normal subjects by comparison with those carrying an adenoma, to examine whether there are pan-colon field effects in addition to local effects. Studies must also establish what the role and interactions of NRP-1 are in the normal colon epithelium in order to establish a clear role for butyrate in the regulation of function as well as homeostasis.

## Materials and methods

### Patient samples and data collection

The study protocol through which samples were acquired has been described elsewhere in detail [39]. Briefly, male patients attending gastroenterology clinics and scheduled for routine endoscopy were recruited to the study. All patients in this study were diagnosed with colorectal adenomas and patients with synchronous pathologies were excluded. Biopsies were collected from the mid-sigmoid (as a conserved landmark between all subjects), from the adenoma and from the contralateral wall to the adenoma (to monitor field effects) during endoscopy. Biopsies were formalin fixed, paraffin embedded (FFPE) and sectioned at multiple levels. A gastrointestinal histopathologist examined all sample, confirming the absence of co-incident pathology in the normal mucosa, and that all adenomas exhibit low-grade dysplasia only. Patients also provided a stool sample, which was extracted for SCFA analysis [39]. The stool sample was collected whilst patients experienced normal bowel habit and not during or immediately after laxative preparation for clinic. The study was approved by North Sheffield Research Ethics Committee (REF: 06/Q2308/93).

### Immunohistochemistry (IHC) for NRP-1 and CgA

NRP-1 and CgA were stained in serial sections to enable analysis of co-localisation of the two factors. Antigen retrieval was performed using heat induced epitope retrieval using a microwave oven, with citrate buffer (pH6) for NRP-1 and DAKO Target Retrieval Solution (DAKO) for CgA. For NRP-1 staining a polyclonal rabbit anti-human NRP-1 antibody (Santa Cruz) and for CgA staining a monoclonal mouse anti-CgA antibody (DAKO) were used. A standard horse-radish peroxidise staining procedure was performed for both antibodies, using biotinylated antibodies (Vector Laboratories, Peterborough) followed by the elite ABC kit (Avidin:Biotinylated enzyme complex; Vector laboratories) and DAB as the chromogen substrate (Vector laboratories) for visualisation. Sections of normal mucosa from the landmark site and adenoma field (contralateral wall) were scored as the percentage of positively stained cells per hemi-crypt for each marker. Only well-orientated hemi-crypts were scored, up to a maximum of 10/section. To assess the colocalisation of NRP-1 and CGA, staining was performed in serial sections and 400 cells classified as GCA^+ ^/NRP^+^, CGA^+ ^/NRP^-^, GCA^- ^/NRP^+^, and CGA^- ^/NRP^-^,. Adenomas were scored for the intensity and percentage of positive NRP-1 and positive or negative of CgA stained cells per each section. All staining was scored by an assessor (DY) blinded to the cases and trained by the project histopathologist (JPB), and second scored by the project histopathologist. The co-localisation analysis was double scored by two assessors (DY & JT), under the supervision of the project pathologist.

### Statistical Analysis

Statistical analysis into the relationship between NRP-1 or CgA staining and faecal butyrate was conducted using SPSS v18 software (Chicago, IL, USA). As the continuous data were not normally distributed the correlation between faecal butyrate levels and NRP-1 or CgA expression was analysed using Spearman's correlation statistics. A further analysis grouped the samples into tertiles by faecal butyrate and used the nonparametric Jonckheere-Terpstra test for ordinal categorical groupings. Data were considered statistically significant at the level of *p *< 0.05.

## Competing interests

The authors declare that they have no competing interests.

## Authors' contributions

DCWY undertook all laboratory work, preliminary statistical analysis and wrote the first draft of the paper; JPB directed the development of IHC methods and directed statistical analysis, co-wrote and edited the manuscript; JT undertook the colocalisation analysis; JSW supervised laboratory work and co-conceived the study; CAS co-conceived the study, undertook statistical analyses, co-wrote and edited the manuscript; BMC conceived the study, directed the work and co-wrote and edited the manuscript. All authors read and approved the final manuscript.
